# Development of a chub mackerel with less-aggressive fry stage by genome editing of arginine vasotocin receptor V1a2

**DOI:** 10.1038/s41598-023-30259-x

**Published:** 2023-02-23

**Authors:** Hirofumi Ohga, Koki Shibata, Ryo Sakanoue, Takuma Ogawa, Hajime Kitano, Satoshi Kai, Kohei Ohta, Naoki Nagano, Tomoya Nagasako, Seiichi Uchida, Tetsushi Sakuma, Takashi Yamamoto, Sangwan Kim, Kosuke Tashiro, Satoru Kuhara, Koichiro Gen, Atushi Fujiwara, Yukinori Kazeto, Takanori Kobayashi, Michiya Matsuyama

**Affiliations:** 1 Aqua-Bioresource Innovation Center (ABRIC) Karatsu Satellite, Faculty of Agriculture, Kyushu University, Saga, 847-0132 Japan; 2 Laboratory of Marine Biology, Faculty of Agriculture, Kyushu University, Fukuoka, 819-0395 Japan; 3 Fishery Third Group, Marine Fisheries Research and Development Center, Japan Fisheries Research and Education Agency (FRA), Kanagawa, 221-8529 Japan; 4 Laboratory of Aquaculture, Faculty of Agriculture, University of Miyazaki, Miyazaki, 889-2192 Japan; 5 Human Interface Laboratory, Factory of Information Science and Electrical Engineering, Kyushu University, Fukuoka, 819-0395 Japan; 6 Molecular Genetics Laboratory, Graduate School of Integrated Sciences for Life, Hiroshima University, Hiroshima, 739-8526 Japan; 7 Laboratory of Molecular Gene Technics, Faculty of Agriculture, Kyushu University, Fukuoka, 812-8581 Japan; 8 Planning and Coordination Department, Fisheries Technology Institute, FRA, Nagasaki, 851-2213 Japan; 9 Aquatic Breeding Division, Aquaculture Research Department, Fisheries Technology Institute, FRA, Mie, 516-0193 Japan; 10 Fisheries Technology Institute, Minamiizu Field Station, FRA, Shizuoka, 415-0156 Japan; 11 Aquatic Breeding Division, Aquaculture Research Department, Fisheries Technology Institute, FRA, Kanagawa, 236-8648 Japan; 12 ABRIC, Faculty of Agriculture, Kyushu University, Fukuoka, 819-0395 Japan

**Keywords:** Biotechnology, Developmental biology, Molecular biology, Physiology

## Abstract

Genome editing is a technology that can remarkably accelerate crop and animal breeding via artificial induction of desired traits with high accuracy. This study aimed to develop a chub mackerel variety with reduced aggression using an experimental system that enables efficient egg collection and genome editing. Sexual maturation and control of spawning season and time were technologically facilitated by controlling the photoperiod and water temperature of the rearing tank. In addition, appropriate low-temperature treatment conditions for delaying cleavage, shape of the glass capillary, and injection site were examined in detail in order to develop an efficient and robust microinjection system for the study. An arginine vasotocin receptor V1a2 (*V1a2*) knockout (KO) strain of chub mackerel was developed in order to reduce the frequency of cannibalistic behavior at the fry stage. Video data analysis using bioimage informatics quantified the frequency of aggressive behavior, indicating a significant 46% reduction (P = 0.0229) in the frequency of cannibalistic behavior than in wild type. Furthermore, in the *V1a2* KO strain, the frequency of collisions with the wall and oxygen consumption also decreased. Overall, the manageable and calm phenotype reported here can potentially contribute to the development of a stable and sustainable marine product.

## Introduction

The world's population has already exceeded 7 billion and is estimated to reach 9.8 billion by 2050^1^. Owing to this rapid population growth, the per capita consumption of animal protein has been increasing on a global scale. By 2030, the protein supply may not be able to keep up with the demand, resulting in a food "Protein Crisis". With regard to aquaculture products especially, competition for acquisition has been predominant worldwide, due to economic growth in emerging countries and the rapid increase in demand for fish and shellfish among the health-conscious population in Europe and the United States. Under such circumstances, the global capture fisheries production has remained flat at around 90 million tons since the 1990s and has almost reached its limit^2^. However, aquaculture production is still on the rise, and in 2014, that of edible fish and shellfish exceeded the catch from natural sources^2^. Thus, the aquaculture industry is expected to continue to expand dramatically as a growing industry in protein supply and contribute significantly to the world's sustainable food supply. For further development of the aquaculture industry, it would be necessary to breed fish, improve them genetically, and produce varieties with traits that are convenient for aquaculture production and suit the consumer tastes.

For the breeding of edible fish, fast-growing red sea bream (*Pagrus major*) strains have been developed by selective breeding^3^. Rainbow trout (*Oncorhynchus mykiss*)^4^ and olive flounder (*Paralichthys olivaceus*)^5^, with disease-resistant traits, have been developed by selective breeding using genetic markers. However, the production of new breeds requires an enormous amount of time, along with a breeding facility specific for a kind of fish, besides a large amount of labor and funds; therefore, breeding of fish has been significantly delayed compared to that of livestock animals. Genome editing technology, which has been introduced recently in practical applications, has been attracting worldwide attention for its ability to artificially induce the desired trait with high accuracy and accelerate breeding remarkably. Genome editing has already been applied to the breeding of edible fish, such as common carp (*Cyprinus carpio*)^6^, channel catfish (*Ictalurus punctatus*)^7^, and red sea bream^8^, with increased muscle mass arising from the editing of myostatin gene that regulates muscle development.

Chub mackerel (*Scomber japonicus*) is a marine fish of the order Perciformes that inhabits temperate and subtropical regions of the world and is an important fish species in Japan. In recent years, aquaculture of this species has been initiated in various areas of Japan for a stable supply that is not affected by the catch from nature; we had established a full life-cycle aquaculture system for the first time in 2012. Besides being a promising species for aquaculture, the chub mackerel is a member of the order Scombridae that includes many important fish species, such as bluefin tuna (*Thunnus orientalis*), yellowfin tuna (*Thunnus albacares*), and Japanese Spanish mackerel (*Scomberomorus niphonius*); therefore, the findings obtained from research on this species may eventually be applicable to many species. In addition, it has useful characteristics for being a model species of marine farmed fish, such as maturation within a year, contrary to other marine fish species that take several years to mature. On the other hand, a problem with the full life-cycle aquaculture of chub mackerel is the low survival rate of fry. Scombridae fish, including bluefin tuna, are known to show aggressive behavior, and even cannibalism in the fry stage^9^, and in chub mackerel, production efficiency is significantly low due to the cannibalistic behavior of the fry. Chub mackerel fry become intensely cannibalistic from approximately 14 days post-hatching, and with usual breeding techniques, only approximately 10% survive immediately after hatching until they grow to a body length of approximately 10 cm. If the survival rate can be improved by suppressing cannibalism, it would be a great advantage for producers, thereby contributing to the improvement of productivity and realization of a sustainable aquaculture system.

The neuropeptides arginine vasotocin (AVT) and arginine vasopressin are key modulators of affiliation and aggression, respectively, in non-mammalian and mammalian vertebrates^10^. For example, tropical damselfish (*Stegastes leucostictus*) tend to have significantly higher aggression against intruders into the territory in the AVT-injected group compared to that in controls^11^. Similarly, administration of AVT has been reported to enhance the aggressive behavior in mudskippers (*Periophthalmus modestus*)^12^. Teleosts have four AVT receptors (V1a1, V1a2, V2a, and V2b)^13^. In a study on bluehead wrasse (*Thalassoma bifasciatum*), administration of a V1a-type receptor antagonist was reported to significantly reduce aggression^14^. In particular, recent studies using genome editing technology have reported the mate-guarding behavior, including aggression, to be significantly reduced in medaka (*Oryzias latipes*) with knocked out V1a2 receptors^15^.

The current study aimed to develop an easy-to-rear manageable chub mackerel variety with reduced cannibalistic behavior during the fry period using genome-editing technology. To conduct genome editing and efficient egg collection, a system with high reproducibility utilizing the stable microinjection method would need to be built. Therefore, we first developed a platform for genome editing in chub mackerel, followed by the development of a *V1a2* knockout line; the frequency of aggression against other fish was quantified by movie data analysis using bioimage informatics, and the trait was evaluated by comparison with that of the wild type.

## Results

### Establishment of a fertilized egg collection system

**Figure 1:**
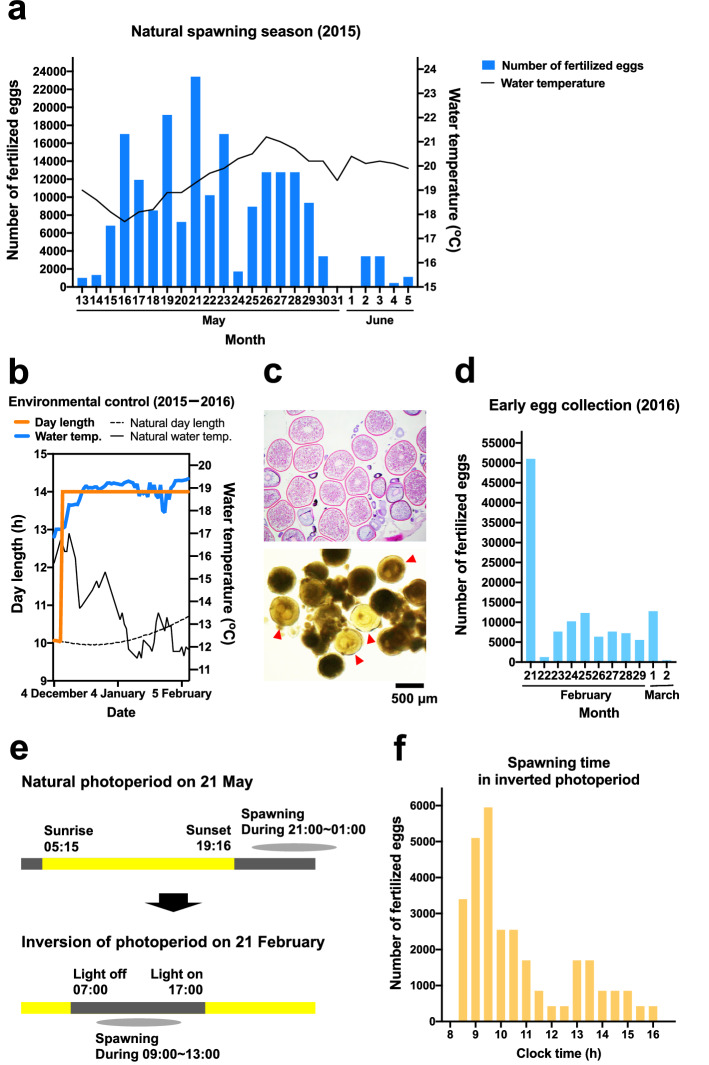
Collection system for fertilized eggs of chub mackerel. (**a**) Results of fertilized egg collection during the natural spawning season of 2015. The blue column shows the number of fertilized eggs collected each day. The bar graph shows the fluctuation of sea water temperature during the period. (**b**) Environmental conditions for artificial maturation. (**c**) Ovarian section of females artificially matured by environmental control (top) and in-vitro bioassay results with human chorionic gonadotropin addition (bottom). Ovarian samples were fixed in Bouin’s solution overnight, dehydrated in a series of ethanol solutions (up to 100%), embedded in paraffin, sectioned at 6 μm using an HM 355S microtome (Thermo Fisher Scientific, Waltham, MA, USA), stained with hematoxylin, and counterstained with eosin. The shredded ovary pieces were transferred to L15 medium (Thermo Fisher Scientific) containing 100 IU of human chorionic gonadotropin (ASKA Pharmaceutical, Tokyo, Japan) and cultured at 18.5 °C for 24 h. Red arrows indicate eggs with germinal vesicle breakdown. (**d**) The number of fertilized eggs collected when spawning was induced in the winter of 2016. Early egg collection was performed by adjusting the environment. (**e**) Spawning time of chub mackerel under natural (upper bar) or artificially reversed day-night photoperiod (lower bar). Black and yellow bars represent night and day times, respectively. (**f**) Time at which fertilized egg was obtained, with day and night of the spawning tank reversed.

Synthetic gonadotropin-releasing hormone analog (GnRHa) was intramuscularly administered to 15 females and 20 males, as reported in previous studies^16^. Chub mackerel actively spawned between 2100 and 0100 h, and the fertilization window for the ovulated eggs of chub mackerel lasts only a few hours^17^. Spawning was monitored every 30 min, and most of the fertilized eggs recovered were at the 1-cell stage. The number of eggs laid varied from day to day, and we were able to obtain more than 20,000 fertilized eggs at the most in one night, and at least approximately 1000 fertilized eggs could be secured every night (Fig. 1a). The amount of spawning was high in the first half of the monitoring period and decreased in the latter half (Fig. 1a). The water temperature during monitoring ranged between 17.7 and 21.2 °C.

Next, we extended the spawning period. Chub mackerel is a seasonal breeding fish, and in the East China Sea near Kyushu Island, multiple spawning occurs in spring to early summer, when the seawater temperature is in the range 16–22 °C^18^. Therefore, we attempted to shift the spawning season by adjusting the environment. In the middle of winter, sexually immature chub mackerel parent fish were reared in an environment-adjustable tank, placed in a long-day photoperiod with 14-h light and 10-h dark period, and the water temperature was heated to the spawning-season temperature (Fig. 1b, details described in “Methods” section). More than 80% of the females were found to possess eggs with complete vitellogenesis in early February, when the water temperature would normally be the lowest in the year (Fig. 1c, upper). Germinal vesicle breakdown was induced in the eggs in an in-vitro bioassay with human chorionic gonadotropin addition, indicating that they have final oocyte maturation potential (Fig. 1c lower). After administering GnRHa to 14 artificially matured females and 16 males on February 20, 2016, spawning was confirmed the next day (Fig. 1d). Fertilized eggs were obtained daily during the 11 days of monitoring (Fig. 1d).

Since early egg collection used a tank surrounded by a blackout curtain to control the environment, we attempted to change the circadian rhythm of chub mackerel by reversing the light–dark cycle from the natural environment (Fig. 1e). After one week of photoperiod-reversal, fertilized eggs could be obtained during the daytime from the first day of spawning, implying a successful reversal of the original spawning time (Fig. 1f). Spawning was concentrated between 2 and 3 h after the lights were turned off, which probably corresponded to 2100 and 2200 h in the evening at normal spawning time (Fig. 1f).

### Establishment of a microinjection system

**Figure 2:**
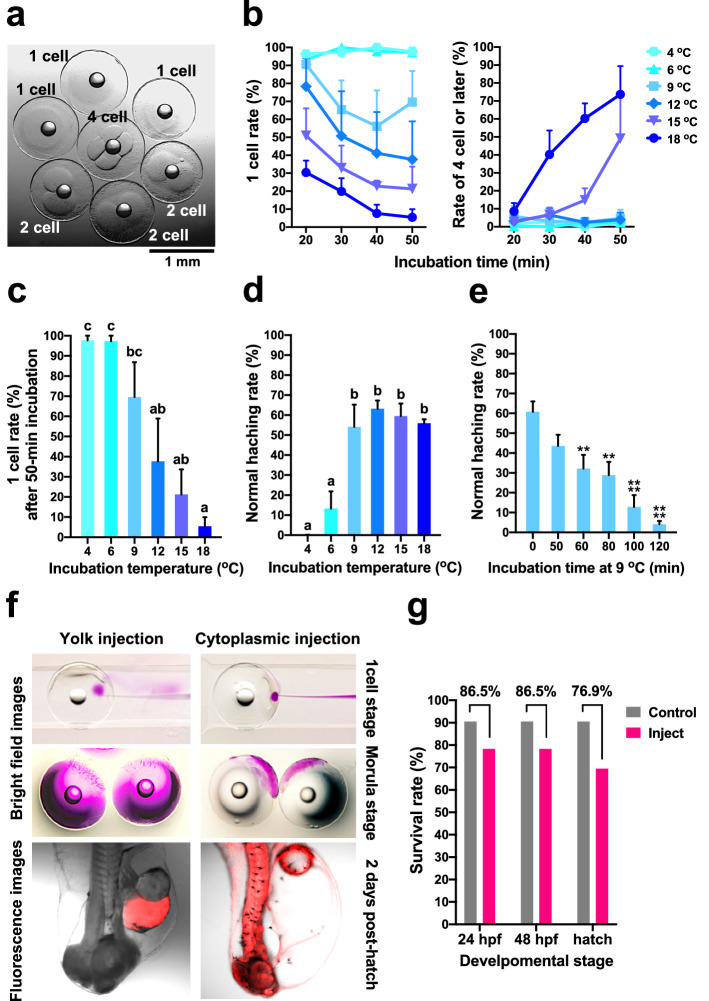
Microinjection into chub mackerel embryos. (**a**) Cleavage of fertilized eggs of chub mackerel. (**b**) Percentage of 1-cell stage eggs at each time (left) and percentage of eggs that reached the 4-cell stage or later (right) when the collected fertilized eggs were incubated in seawater at various temperatures (n = 3). (**c**) Percentage of 1-cell stage eggs when the collected fertilized eggs were placed in seawater at various temperatures for 50 min. Different letters above the bars represent significant differences (P < 0.05) in data across various incubation temperatures. (**d**) Normal hatching rate, when the collected fertilized eggs were placed in seawater at various temperatures for 50 min and then returned to the natural seawater temperature of 18 °C for incubation. Different letters above the bars represent significant differences (P < 0.05) in data across various incubation temperatures. (**e**) Normal hatching rate, when the collected fertilized eggs were placed in seawater at 9 °C for various durations from 50 to 120 min and then returned to the natural seawater temperature of 18 °C for incubation. Asterisks indicate a significant difference when compared to the control group. **P < 0.01, ****P < 0.0001. (**c**–**e**) Data are expressed as the mean ± SEM (n = 3) and analyzed by one-way ANOVA followed by a Turkey’s multiple comparison test. (**f**) Localization of the dye at each stage of development when the fluorescent dye (200 mM KCl solution containing 0.05% Dextran, Rhodamine B) was microinjected into the yolk or cytoplasm. (**g**) Embryo survival and normal hatchability after 24 and 48 h of microinjection into the cytoplasm.

Fertilized chub mackerel eggs began cell division (cleavage) approximately 50–60 min after fertilization, and then divided every 10–15 min (Fig. 2a). Delaying the cleavage would be essential since that is often found to have begun by the time fertilized eggs were collected and moved to the laboratory to prepare for injection. Collected fertilized eggs were transferred to Petri dishes filled with seawater at various temperatures from 18 °C (natural water temperature) to 4 °C. Approximately 30 eggs were randomly picked up every 10 min between 20 and 50 min at each temperature and microscopically examined for the proportion of eggs in the 1-, 2-, and 4-cell stages. At each time point, the lower the water temperature, the higher was the proportion of 1-cell stage eggs (Fig. 2b). However, in the eggs incubated at natural water temperature, the ratio of 1-cell stage eggs to all checked eggs was approximately 30% after 20 min and less than 10% after 50 min (Fig. 2b). The proportion of eggs that developed after the 4-cell stage increased as the water temperature increased, and at natural water temperature, it was 40% after 30 min and 70% or more after 50 min (Fig. 2b). The percentage of 1-cell stage eggs after 50 min was significantly higher in eggs incubated in seawater below 9 °C compared to that in those incubated at natural water temperature (Fig. 2c). After 50 min of egg collection, in seawater at 9 °C, approximately 70% of eggs remained in the 1-cell stage, whereas at 4 °C and 6 °C, more than 90% of eggs remained in the 1-cell stage (Fig. 2c). There was no significant difference in the percentage of 1-cell stage eggs across the three temperatures (4, 6, and 9 °C; Fig. 2c). Eggs incubated for 50 min at each temperature were eventually returned to the natural water temperature of 18 °C, and normal hatchability was examined. In the incubation groups at 18 °C and 9 °C, the normal hatching rate was approximately 60%, and there was no difference between different temperatures. On the other hand, eggs incubated in seawater at 4 °C and 6 °C hardly hatched and stopped developing midway (Fig. 2c). Therefore, when the eggs were incubated at a low temperature of 6 °C or lower, development was stopped rather than delayed. By stocking the collected eggs in seawater at 9 °C, cleavage could be delayed without significantly affecting the normal hatching rate. Next, we investigated the storage time of the collected eggs in seawater at 9 °C. After collection, the fertilized eggs were immediately transferred to seawater at 9 °C, incubated for 50–120 min, moved to the normal water temperature of 18 °C, and examined for their hatching rate. When stocked in seawater at 9 °C for 60 min or longer, the normal hatching rate was significantly reduced compared to that of eggs at natural water temperature (Fig. 2e). When the eggs were stocked for 120 min in seawater at 9 °C, they hardly hatched, even when the water temperature returned to normal (Fig. 2e). Therefore, the duration for which they could be stocked in seawater at 9 °C for delaying cleavage was considered to be 60 min at the most. The percentage of 1-cell stage eggs when incubated in seawater at 9 °C for 50 min was close to 70%, and since injection could be continued while gradually returning to the natural water temperature, the normal injection time could be stretched up to two or three times.

The injection site of the genome editing solution was examined next. A fluorescent dye was injected into the yolk or cytoplasm of the fertilized egg, and transfer of the dye was observed. The dye injected into the yolk remained at the yolk site in both the morula and the fry after hatching (Fig. 2f, left), whereas, the dye injected into the cytoplasm was observed in the embryo in the morula stage, and red fluorescence was observed throughout the body in the fry post-hatching (Fig. 2f, right). This implied that cytoplasmic streaming did not occur in fertilized eggs of chub mackerel, and direct injection into the cytoplasm was necessary.

The glass capillary used for microinjection was sharpened and thinned to a 1-µm diameter, enabling smooth injection administration. Owing to the high internal pressure of chub mackerel eggs, a needle with a small tip diameter would be required to prevent cytoplasmic leakage during injection. Eggs infused with dye into the germ disk showed an 86.5% survival rate at 24 and 48 h compared to untreated eggs (Fig. 2g). The normal hatching rate, compared to that of untreated eggs, was 76.5%, showing a survival rate of approximately 80% (Fig. 2g).

### Construction of *V1a2* knockout strain

**Figure 3:**
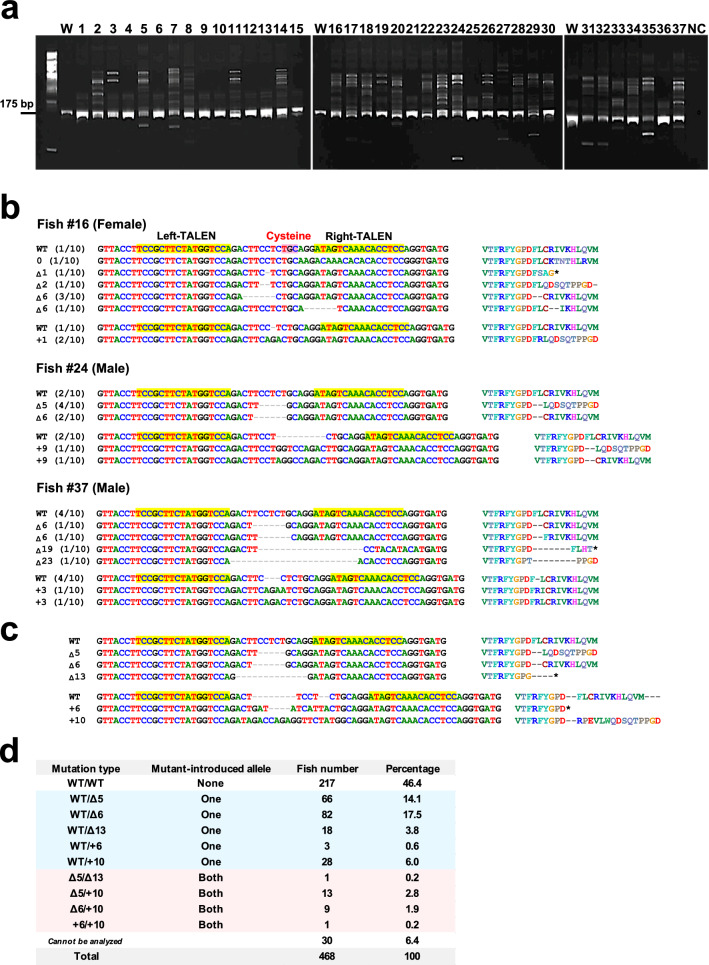
Production of the *V1a2* knockout lineage. (**a**) Results of the heteroduplex mobility assay (HMA) analysis of all *V1a2* KO F_0_ generation that survived 5 months of age. W indicates wild type. NC indicates negative control. (**b**) The gene sequence and amino acid sequence of the target site of the F_0_ generation, which had a particularly high mutagenesis rate and was selected for F_1_ generation production (one female and two males). The label number (#) of the individual matches the number of HMA analysis result in (**a**). Alignment results of the data obtained by sequence analysis of 10 clones of each fish are shown. The left and right TALEN recognition areas are highlighted in yellow. Δ indicates a base deletion, and plus indicates a base insertion. *indicates a stop codon. (**c**) As a result of sequence analysis of the target gene sites of all F_1_ fish that survived 5–6 months post-hatch, three patterns of defective mutation and two patterns of base insertion were confirmed. (**d**) Breakdown of mutants of both alleles in all F_1_ fish sequenced. In 30 individuals, the genotype is still unknown, since clear sequence analysis results were not obtained.

We used transcription activator-like effector nuclease (TALEN) technology to create the *V1a2* knockout strain. Left and right TALEN mRNA, at 100 ng/μl concentration each, were microinjected into the cytoplasm of 1- or 2-cell stage fertilized eggs. After 24 h post-injection, 591 fertilized eggs microinjected with TALEN mRNA survived. Since all the eggs were kept in a fry-breeding tank, the number of normal hatches could not be determined. By the 24th day post-hatch, 52 fry of the *V1a2* KO F_0_ generation survived. At 5 months post-hatch, 37 fish survived, and fins were collected from each fish and genotyped. The heteroduplex mobility assay analysis showed the mutations in the target DNA of all individuals (Fig. 3a). Sequencing analysis was performed and one female and two males with the highest rate of introduction of frameshift mutation were selected for F_1_ generation production (Fig. 3b). In fish, many types of mutations have been reported to be present in a mosaic pattern in F_0_ individuals after genome editing^19^. Many frameshift mutation patterns were observed in the target genomic regions of the three selected fish (Fig. 3b).

The F_1_ generation was produced during the spawning season in 2018, and 468 fish survived after 5–6 months of hatching; the genotypes of all fish were confirmed by sequence analysis. Three types of defective mutations and two types of insertion mutations were identified (Fig. 3c). The 13-base deficiency (Δ13), and 6-base and 10-base insertion (+ 6 and + 10) mutations were variants that could not be confirmed by sequence analysis of the parents of F_0_ generation (Fig. 3b). This was probably because only 10 clones were sequence-analyzed in each F_0_ fish, and more varied mutation patterns could possibly be confirmed by analyzing more samples by next-generation sequencing analysis. Of all the F_1_ strains analyzed, 46.4% were wild-type, 42% were *V1a2*^+*/−*^, which had mutations introduced into one allele, and 5.1% were *V1a2*^*−/−*^, which confirmed the introduction of mutations into both alleles (Fig. 3d).

**Figure 4:**
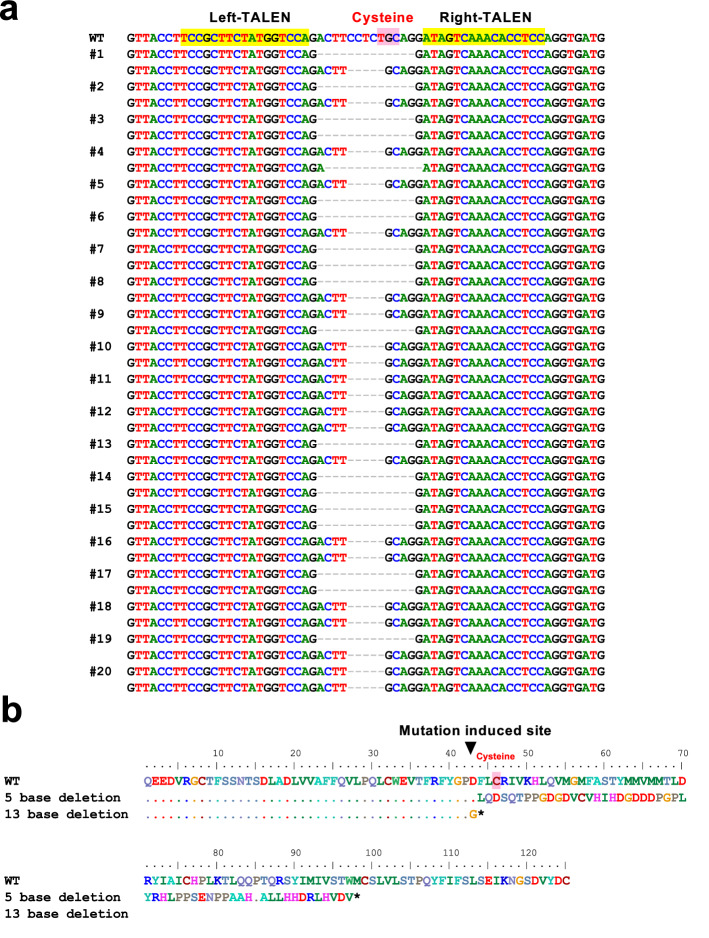
Establishment of the *V1a2* knockout lineage. (**a**) Sequence analysis results of target gene sites in 20 F_3_-generation fry immediately after hatching. Defect mutations of 5 or 13 bases were introduced in both alleles in all individuals, establishing the *V1a2*^−/−^ line. (**b**) Changes in the V1a2 amino acid sequence due to 5-base and 13-base defective mutations were confirmed in the F_3_ generation. The asterisk indicates a stop codon.

In the F_2_ generation, not all fish could be made *V1a2* null; however, in the F_3_ generation produced in 2020, all fish were *V1a2* null. Sequence analysis of the genotypes of 20 F_3_-generation fry, immediately after hatching, revealed defective mutations in both alleles in all individuals (Fig. 4a). The mutation patterns were (Δ5, Δ5), (Δ13, Δ13), and (Δ5, Δ13) null (Fig. 4a); the rates of appearance were 30%, 25%, and 45%, respectively. In both Δ5 and Δ13 defect patterns, cysteine residues that formed the S–S bridge, defined at the target site, were lost due to frameshift (Fig. 4b). In the case of a 5-base deletion, 54 amino acids different from normal V1a2 were estimated to be produced as a result of a frameshift mutation (Fig. 4b). In contrast, in the case of a 13-base deficiency, a stop codon appeared immediately after the mutation introduction site (Fig. 4b). Although the heterozygous KO group had different deletion patterns in both alleles, since the target gene was inactivated in both alleles, it was presumed to be *V1a2* null. In the F_3_ generation fry, the frequency of aggressive behavior was quantified by phenotypic analysis.

### Quantification of the frequency of cannibalistic behavior

**Figure 5:**
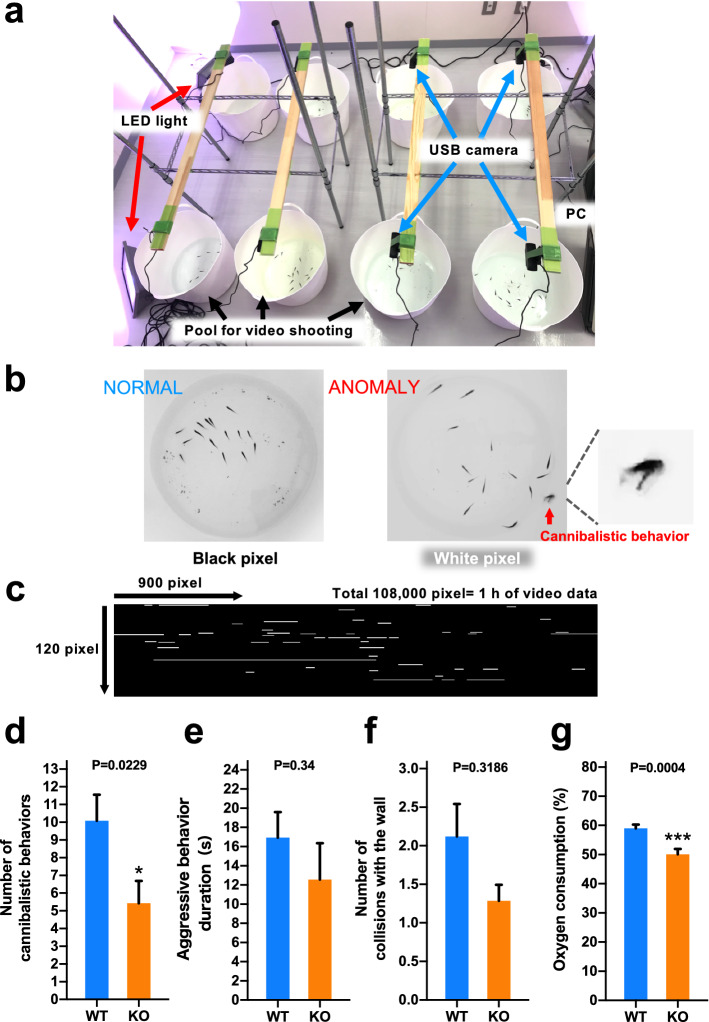
Phenotypic analysis of mutants. (**a**) Video shooting environment for fry. Eight sets of video shooting containers and video cameras were prepared, such that video could be shot simultaneously in all eight containers. A white, smooth, circular container (LB-4139; Nitori, Hokkaido, Japan) with a diameter of 35 cm and high contrast with the color of the back of the fry was used to capture their swimming image. A USB video camera (HD Pro Webcam C920r; Logicool, Tokyo, Japan) with a 30-fps frame rate and 1280 × 720 image resolution, connected to a PC, was attached to the upper part of each pool, and video was recorded. (**b**) Normal swimming behavior of fry (right) and the moment of cannibalistic behavior, in which the group of fry is disturbed (left). (**c**) One example of the analysis result screen of the software; 900 pixels are lined up horizontally and 120 pixels are lined up vertically. One pixel is one frame of a moving image divided into one still image frame every 1/30 s, and 108,000 frames resulted from moving image analysis for 1 h. (**d**) Frequency of cannibalistic behavior per hour. *P = 0.0229. (**e**) Duration of aggression in one cannibalistic behavior. (**f**) The number of collisions with the container wall per hour. (**g**) Oxygen consumption of WT and KO fry over 2 h in the video shooting pools. ***P = 0.0004. Data are expressed as the mean ± SEM [n = 25 (WT) and 21 (KO)] and were analyzed by unpaired two-tailed Student’s t-test followed by an f-test. *WT* wild type, *KO* knockout.

We developed a video content analysis system based on a bioimage-informatics technique for the objective and automatic surveillance of cannibalistic behavior in a fry group. The system comprised of a video camera that would capture a fry group in a pool, and computer software that would analyze the captured video (Fig. 5a). The software detected cannibalistic behaviors as anomalous actions in the fry group. By counting the frequency of anomalous actions for a certain duration (such as one hour), we could evaluate how often cannibalistic behaviors occurred during the period. With the introduction of this software, experimenters could be freed from the hassle of visually checking hours of video data. Furthermore, since the evaluation results did not depend on any subjective observation, reliable evidence could be obtained from the study.

Chub mackerel fry usually swim in groups in the same direction and the software evaluates this swimming status as a feature vector (details described in ‘Supplementary information’). Vectors during cannibalistic behaviors are different from those during normal behaviors since cannibalistic behaviors often show very large and scattered motions of the fry (Fig. 5b). Figure 5c shows an example of the software analysis result screen, which is a two-dimensional representation of each hour of video data. The video data were divided into one still image frame every 1/30 s. One pixel on the result screen represents one frame. That is, one hour's worth of video analysis result was displayed in 108,000 frames. The pixel on the upper left side represents the first frame and the next one to the right represents the second frame. Proceeding 900 frames to the right (30 s) will return to the left edge, go down one step, and proceed right again for 900 frames (30 s). The bottom right is the 108,000th frame, which is 3600 s (1 h) from the start of the video. The time point when a certain cannibalistic behavior occurred can be determined as the timestamp (white pixel) in the video frame where the anomaly feature vector was detected (Fig. 5c).

Behavioral analysis showed that the frequency of cannibalistic behavior per hour was reduced by 46% in the *V1a2* KO group than in the wild type, which was a significant difference (P = 0.0229) (Fig. 5d). The duration of aggression was not significantly different with respect to the wild type (P = 0.34) but was reduced by 26% in the *V1a2* KO group than in the wild type (Fig. 5e). The number of collisions with the wall was also not significantly different with respect to the wild type (P = 0.3186), but it was 39% lower in the *V1a2* KO group than in the WT group (Fig. 5f). Measurement of dissolved oxygen amount in the pool before and after the 2-h video shooting revealed that oxygen consumption was 15% lower in the *V1a2* KO group than in the wild type, which was a significant difference (P = 0.0004) (Fig. 5g).

## Discussion

For genome editing to be performed efficiently in fish, a genome-editing reagent needs to be microinjected into fertilized eggs immediately after fertilization; therefore, efficient collection of fertilized eggs immediately after fertilization is a major technical issue. Our research center (ABRIC) has already developed an efficient fertilized egg collection system and a mutant production system for the Japanese anchovy (*Engraulis japonicus*), a marine teleost fish^20^. In the case of chub mackerel, vitellogenesis progresses sufficiently in females reared in a small land-based tank of several kiloliters; however, final oocyte maturation and ovulation do not occur. However, testicular maturation occurs normally in males. This is because in captive females, although luteinizing hormone (LH), which promotes final oocyte maturation, is sufficiently produced in the pituitary gland, GnRH1, which promotes LH release, is not secreted from the hypothalamus^16^. Therefore, when GnRHa is injected into the back muscles of captive females, final oocyte maturation is induced, and spawning in the tank begins 34–36 h post-administration. When the seawater temperature exceeds 23 °C, egg regression begins and spawning ends^17^. By controlling the breeding environment, the spawning season can be shifted forward and the spawning time can be changed. The chub mackerel genome editing experimental system established here ensures that homogeneous samples are obtained at a date and time that is convenient for the experimenter for approximately half the year.

Injection manipulation should be performed during early embryogenesis, mostly in 1-cell eggs, shortly after spawning and fertilization, or, at the latest, in 2-cell eggs after the first cleavage. Delaying cleavage is of utmost importance since in most cases cleavage has already begun when the fertilized eggs are collected and moved to the laboratory to prepare for injection. By examining the appropriate low-temperature treatment conditions for delaying cleavage, the shape of the capillaries, and the injection site in detail, the time for microinjection could be extended to approximately twice the conventional time. However, egg quality greatly affects genome editing efficiency. In many fish species, older, larger, and more nutritious parents lay high-quality eggs and show a higher fry survival rate^21,22^. Chub mackerel reaches puberty in 1 year; however, a large female over the age of two can potentially lay high-quality eggs^23^. To obtain microinjected eggs for genome editing, consideration of the maternal effects of spawning parents would be important.

To efficiently establish genetically modified animals through genome editing, the selection of individuals with the desired genotype and their breeding across generations are essential. The first factor influencing process difficulty is the germline transmission efficiency of F_0_ mosaic animals. Genotyping results using genomic DNA extracted from the caudal fin suggested that wild-type sequences of the three parental F_0_ generations selected for F_1_ generation production averaged 20% of the ten clones analyzed. On the other hand, genotyping of all F_1_ generation individuals revealed that nearly 50% of the individuals were completely wild type, which was higher than the theoretical estimate. In particular, the proportion of individuals with mutations introduced in both alleles in the F_1_ generation was 5.1%, which was significantly lower than expected. However, since we did not investigate the proportion of genotypes in fertilized eggs or fry immediately after hatching, we cannot yet discuss the efficiency of the passage of mutations to the F_1_ generation in detail. Although we did not evaluate the traits of *V1a2*^+/−^ individuals, there could be some phenotypes with low aggression even in the heterozygous knockouts. Such a phenotype may be disadvantageous in coexistence with wild-type fry, and many *V1a2*^+/−^ and *V1a2*^−/−^ individuals may have been selected together in the study. In fact, in the F_0_ generation of red sea bream, in which a mosaic-like gene mutation was introduced by genome editing, there was no significant difference in the mutation introduction rate and mutation type ratio between the pectoral fins and germ cells^8^. Even in chub mackerel, the efficiency of mutation introduction is unlikely to differ greatly between caudal fin and germ cells. At the present stage, we do not assume that there could be a problem with the stable transmission of mutations to the next generation in our experimental system. A detailed evaluation of the efficiency of mutation introduction into germ cells of the F_0_ mosaic generation would be useful when performing genome editing for various genes in this species in the future.

Phenotypic analysis of the *V1a2* KO strain revealed the frequency of aggressive behavior, including cannibalistic behavior, to be approximately half that of the wild type. In other words, a phenotype that is manageable in the fry stage was successfully obtained by genome editing of *V1a2*, as per the original purpose of this study. The fact that cannibalistic behavior was halved suggested that the survival rate of fry could be more than double. Since survival rate is an important factor for seed production, a detailed comparison of production efficiencies between wild-type and *V1a2* mutants would need to be repeated over the next few years. In addition, since the mutant has a gentler personality than the wild type, a detailed evaluation of growth rate and feed efficiency would also be necessary. Interestingly, an unexpected phenotype other than the reduced frequency of cannibalism may have been present. Although there was no significant difference, the frequency of collisions with the wall of the container tended to decrease by approximately 40% owing to the gentle trait. Death due to collision with the walls of sea cages is a problem for tuna, which is a species closely related to chub mackerel, owing to its high swimming speed; research is being conducted currently for the development of mutants with low agility^24^. Gentle traits may also contribute to a reduction in collision mortality at aquaculture sites. In addition, the *V1a2* mutant strain was also suggested to consume less oxygen than the wild-type strain. Although the reason for this is still unknown, the amount of active swimming is considered to be lesser in the mutant than in the wild type. Catastrophic damage to aquaculture due to red-tides has been reported worldwide^25,26^. Although the detailed mechanism of red-tide death is still unknown, overgrown causative algae are considered to deprive fish of their ability to carry oxygen in their blood, as a result of which, the fish struggle and become oxygen-deficient to avoid red-tides^27^. The *V1a2* mutant strain could possibly be more resistant to hypoxic conditions, such as when red tide occurs, than the wild-type. In the future, various advantages of this breeding method will be clarified by conducting more multifaceted trait evaluations.

In this study, an experimental system that enables efficient egg collection and genome editing in chub mackerel was developed. Chub mackerel fry, whose genome was edited in *V1a2*, had half the cannibalistic behavior of the wild type. Breeding the gentle and manageable trait would be extremely beneficial for the domestication and efficient production of wild fish. On the other hand, although we succeeded in producing a chub mackerel strain with a gentle personality, we did not investigate whether the survival rate improved, and further research is needed in the future. We hope that the implementation of this breed will contribute to the development of stable and sustainable marine product production.

## Methods

### Animals and spawning induction

In May 2015, in order to obtain fertilized eggs during the normal spawning season, 2-year-old fish purchased from a fish farm in Oita Prefecture were transferred to the Fishery Research Laboratory, Kyushu University, and moved into a 3-kL concrete tank with running seawater. For the early egg collection experiment, 2-year-old fish were purchased from a fish farm in Saga Prefecture in December 2015 and transferred to the ABRIC Karatsu satellite, Kyushu University. For spawning induction, a GnRHa [des-Gly10-(D-Ala6) LHRH ethylamide, 400 μg/kg body weight; Sigma-Aldrich, St. Louis, MO, USA], suspended in cocoa butter, was injected into the back muscle of the fish. The spawn eggs were collected using a net attached to the drain outlet of the spawning tank. Since fertilized eggs float on the surface of seawater while unfertilized eggs sink, the collected eggs were transferred to a measuring cylinder and allowed to stand for a while to separate the fertilized eggs from the unfertilized ones. Finally, the floating fertilized eggs were collected and counted.

### Artificial maturation of immature parent fish

On December 4, 2015, fish were moved to a 5-kL fiber-reinforced plastic (FRP) tank with a blackout curtain. Five 760 lm LED bulbs were installed on the ceiling of the blackout curtain and adjusted for a long-day photoperiod (LD, 14L:10D). The lights were adjusted, using a timer, to turn on at 0500 and turn off at 1900 h. To prevent collisions with the wall at night, a small LED bulb (45 lm) was installed as a nightlight. Brightness near the water surface was approximately 400 lx for all lights. The water temperature was adjusted between 18 and 19 °C, corresponding to that during the spawning season, using a titanium coil heater. To obtain fertilized eggs during the daytime, the photoperiod was changed one week before the induction of spawning, such that lights turned off at 0700 and turned on at 1700 h.

### Molecular cloning of *V1a2*

Polymerase chain reaction (PCR) was performed using a cDNA library prepared from the brain, as a template, to obtain a partial sequence of *V1a2* from the deduced 1st exon. Thermal cycling consisted of an initial denaturation at 95 °C for 9 min, followed by 35 cycles at 95 °C for 15 s, 50 °C for 15 s, and 72 °C for 30 s. The polymerase used was that included in AmpliTaq Gold 360 Master Mix (Thermo Fisher Scientific, Waltham, MA, USA), and the content of the reaction mix was in accordance with the manufacturer’s instructions. Sequences of the primers used are as follows: Fw, 5ʹ-TGGTCGCCTTCTTCCAGGTGCTAC-3ʹ and Rv, 5ʹ-CCAATCATCCCGTTCTTACTCGCAG-3ʹ.

### Construction of TALEN plasmids and in-vitro transcription

Platinum TALEN plasmids were constructed using the Platinum Gate TALEN Kit (Addgene; Kit #1000000043), as described previously^28^; ptCMV-153/47-VR was a destination vector. The TALEN pair was designed to target the S–S binding site, which retained the conformation of V1a2. The target sequences of TALENs are as follows: left, 5ʹ-TTCCGCTTCTATGGTCCA-3ʹ and right, 5ʹ-TGGAGGTGTTTGACTAT-3ʹ. To prepare TALEN mRNAs, the left and right TALEN plasmids were linearized with *Sma*I and purified using a QIAquick PCR Purification Kit (Qiagen, Aarhus, Denmark). mRNAs were synthesized and purified using the mMESSAGE mMACHINE T7 ULTRA Kit (Thermo Fisher Scientific) and RNeasy Mini Kit (Qiagen), respectively, according to the manufacturer’s instructions.

### Microinjection

Microneedles were created using a Micropipette Puller PC-10 (Narishige, Tokyo, Japan) to stretch a glass tube with a core (GD-1; Narishige). Using a micro grinder EG-400 (Narishige), the tip diameter was thinned to about 1 μm, and the needle tip was sharpened to an acute angle of 20°. Collected fertilized eggs were carefully arranged in the grooves of a 1.5% agarose hardened in a 15-cm diameter plastic Petri dish under a stereomicroscope and filled with filtered seawater. Next, 200 mM KCl solution containing 0.05% (w/v) dextran and rhodamine B (Thermo Fisher Scientific) was injected, and localization of the dye at each developmental stage was investigated. Images of bright-field eggs were captured using a Nikon DS-Fi2 camera (Nikon, Tokyo, Japan) mounted on a stereomicroscope SZX7 (Olympus, Tokyo, Japan). Fluorescence images were obtained using a LSM-700 confocal laser-scanning microscope (Carl Zeiss, Jena, Germany).

### F_0_ founder production

The F_0_ generation was produced on May 4th and 5th and June 16, 2017. Shortly after spawning, fertilized eggs were collected and pre-incubated at 9 °C in filtered seawater to delay embryonic development until microinjection. Before injection, the TALEN mRNA solution was diluted to a concentration of 100 ng/μl using 200 mM KCl solution, and phenol red (Nacalai Tesque, Kyoto, Japan) was added to a final concentration of 0.25% for visualization during microinjection. TALEN mRNA was injected into the germ disks of 1- and 2-cell stage eggs, transferred to sterilized seawater in a 5-l glass chamber, and incubated at 18 °C. Microinjection was performed for three days and 591 eggs survived 24 h after injection. The surviving eggs were moved to a 100-l polycarbonate tank.

### Genotyping analysis and mating

Fish were anesthetized with 200 ppm of 2-phenoxyethanol (FUJIFILM Wako Pure Chemical Corporation, Osaka, Japan), and a part of the caudal fins was cut with sharp scissors. All individuals were identified by inserting a PIT tag (BIO8; Biomark, Boise, ID, USA) into the back muscle. To detect indel mutations, a heteroduplex mobility assay was performed, and amplicons containing the target site were sequenced. Fins were lysed in alkaline solution (25 mM NaOH, 0.2 mM EDTA) for 5 min at 98 °C and neutralized with a neutralizer (1 M Tris–HCl, pH 6.8). A short fragment, including the target site of the TALENs, was amplified by PCR. Sequences of the primers used are as follows: Fw, 5ʹ-CACCTCAGACCTGGCCGAC-3ʹ and Rv, 5ʹ-ACGGTCCAGGGTCATCATCAC-3ʹ. The resulting amplicons were electrophoresed on a 15% polyacrylamide gel (Fujifilm). The PCR product was purified, ligated to the pGEM-T easy vector (Promega, Madison, WI, USA), transformed into JM109 competent cells (Takara, Shiga, Japan), and the latter spread on agar medium. The obtained colonies were randomly picked, PCR performed, and approximately 10 transformed colonies sequenced (Eurofins Genomics Japan, Tokyo, Japan). Samples were prepared according to the manufacturer’s instructions. Multiple alignments of gene sequences were performed with the Molecular Evolutionary Genetics Analysis (MEGA) X software (Version 10.1.8) using the ClustalW algorithm (https://www.megasoftware.net) of MEGA X. The selected individuals were administered GnRHa and allowed to spawn spontaneously in 3- or 5-kL land-based indoor tanks for next-generation production.

### Behavior analysis

Behavior analysis was performed using F_3_-generation fry (22 days post-hatch; average body length 29.04 ± 1.05 mm; average body weight 0.27 ± 0.03 g), in which *V1a2* was knocked out in both alleles. Comparative WT fry used were aged 20 days post-hatch, with an average body length of 31.6 ± 1.13 mm and an average body weight of 0.28 ± 0.03 g. Fry raised in a 500-l polycarbonate tank were gently scooped up with a net and placed in a container containing 5 l of seawater (Fig. 5a). Water depth was approximately 15 cm. Since aeration cannot be performed during video shooting, oxygen was slowly injected into the pool, for approximately 20 min, so that the dissolved oxygen content of the water tank was 15 mg/l to prevent oxygen deficiency. Since the analysis software could only track up to 15 fry, ten relatively large and five relatively small fry were visually selected for the experiment, ensuring the possibility of fights. Windows in the room were covered with a blackout curtain to prevent outside light from entering, and the room temperature was set at 19 °C, mimicking the natural seawater temperature. To prevent the reflection of fluorescent light on the water surface of the pool, four LED floodlights (LD106; Good Goods, Osaka, Japan) were illuminated on the white wall (Fig. 5a). The video was shot for 120 min in each pool; the first 30 min was acclimatization time, and the last 30 min were for observing the weakened fish; the remaining 60-min video data were used for analysis. The video was recorded for four consecutive days, and video data for analysis were obtained for a total of 25 h in the WT group and 21 h in the KO group, corresponding to approximately 1 day in a fry breeding tank. In the KO group, we lost 4 h video data due to unexpected data corruption. The amount of dissolved oxygen in the pool was measured in all containers at the beginning and at the end of imaging using a DO meter (YSI ProSolo; Xylem, Kanagawa, Japan), and the oxygen consumption was calculated. Animal experiments were performed according to the guidelines for animal experiments at Kyushu University, and the experimental protocols were approved by the Animal Experiments Committee of Kyushu University. Our manuscript confirms that the study has been reported in accordance with ARRIVE guidelines (https://arriveguidelines.org).

### Computer software for video data analysis

The detailed principle of abnormal behavior detection is explained in the supplementary file.

### Statistical analysis

Prism 8 (GraphPad Software, San Diego, CA, USA) was used for graph creation and data analysis.

## Supplementary Information

Supplementary Information.

## Data Availability

The sequence information generated and analyzed during the current study are available in the DNA Data Bank of Japan (DDBJ) repository [Accession number: LC722364].

## References

[1] United Nations. Department of Economic and Social Affairs, Population Division (2019). World Population Prospects 2019: Highlights, ST/ESA/SER.A/423 (2019).

[2] FAO. The State of World Fisheries and Aquaculture 2018—Meeting the sustainable development goals. Rome. Licence: CC BY-NC-SA 3.0 IGO (2018).

[3] Murata O (1996). Selective breeding for growth in red sea bream. Fish. Sci..

[4] Ozaki A (2001). Quantitative trait loci (QTLs) associated with resistance/susceptibility to infectious pancreatic necrosis virus (IPNV) in rainbow trout (*Oncorhynchus mykiss*). Mol. Genet. Genom..

[5] Fuji K (2007). Marker-assisted breeding of a lymphocystis disease-resistant Japanese flounder (*Paralichthys olivaceus*). Aquaculture.

[6] Zhong Z (2016). Targeted disruption of sp7 and myostatin with CRISPR-Cas9 results in severe bone defects and more muscular cells in common carp. Sci. Rep..

[7] Khalil K (2017). Generation of myostatin gene-edited channel catfish (*Ictalurus punctatus*) via zygote injection of CRISPR/Cas9 system. Sci. Rep..

[8] Kishimoto K (2018). Production of a breed of red sea bream *Pagrus major* with an increase of skeletal muscle mass and reduced body length by genome editing with CRISPR/Cas9. Aquaculture.

[9] de la Serna Sabate F (2010). Onset and development of cannibalistic and schooling behavior in the early life stages of Pacific bluefin tuna *Thunnus orientalis*. Aquaculture.

[10] Goodson JL, Bass AH (2001). Social behavior functions and related anatomical characteristics of vasotocin/vasopressin systems in vertebrates. Brain Res. Rev..

[11] Santangelo N, Bass AH (2006). New insights into neuropeptide modulation of aggression: Field studies of arginine vasotocin in a territorial tropical damselfish. Proc. R. Soc. B..

[12] Kagawa N (2013). Potential roles of arginine-vasotocin in the regulation of aggressive behavior in the mudskipper (*Periophthalmus modestus*). Gen. Comp. Endocrinol..

[13] Yamaguchi Y (2012). The fifth neurohypophysial hormone receptor is structurally related to the V2-type receptor but functionally similar to V1-type receptors. Gen. Comp. Endocrinol..

[14] Semsar K, Kandel FLM, Godwin J (2001). Manipulations of the AVT system shift social status and related courtship and aggressive behavior in the bluehead wrasse. Horm. Behav..

[15] Yokoi S (2015). An essential role of the arginine vasotocin system in mate-guarding behaviors in triadic relationships of medaka fish (*Oryzias latipes*). PLoS Genet..

[16] Shiraishi T (2008). Time course of final oocyte maturation and ovulation in chub mackerel *Scomber japonicus* induced by hCG and GnRHa. Fish. Sci..

[17] Shiraishi T (2005). Reproductive parameters of the chub mackerel *Scomber japonicus* estimated from human chorionic gonadotropin-induced final oocyte maturation and ovulation in captivity. Fish. Sci..

[18] Yukami R, Ohshimo S, Yoda M, Hiyama Y (2009). Estimation of the spawning grounds of chub mackerel *Scomber japonicus* and spotted mackerel *Scomber australasicus* in the East China Sea based on catch statistics and biometric data. Fish. Sci..

[19] Ansai S, Kinoshita M (2014). Targeted mutagenesis using CRISPR/Cas system in medaka. Biol. Open.

[20] Sakaguchi K (2019). Comprehensive experimental system for a promising model organism candidate for marine teleosts. Sci. Rep..

[21] Hixon MA, Johnson DW, Sogard SM (2014). BOFFFFs: On the importance of conserving old-growth age structure in fishery populations. ICES J. Mar. Sci..

[22] Green BS (2008). Maternal effects in fish populations. Adv. Mar. Biol..

[23] Yoneda M (2022). Maternal spawning experience and thermal effects on offspring viability of chub mackerel and their influence on reproductive success. Front. Mar. Sci..

[24] Higuchi K (2019). Targeted mutagenesis of the ryanodine receptor by Platinum TALENs causes slow swimming behaviour in Pacific bluefin tuna (*Thunnus orientalis*). Sci. Rep..

[25] Elbrächter M (1998). Exotic flagellates of coastal North Sea waters. Helgol. Mar. Res..

[26] Armijo J, Oerder V, Auger PA, Bravo A, Molina E (2020). The 2016 red tide crisis in southern Chile: Possible influence of the mass oceanic dumping of dead salmons. Mar. Pollut. Bull..

[27] Kim CS, Lee SG, Kim HG (2000). Biochemical responses of fish exposed to a harmful dinoflagellate *Cochlodinium polykrikoides*. J. Exp. Mar. Biol. Ecol..

[28] Sakuma T (2013). Repeating pattern of non-RVD variations in DNA-binding modules enhances TALEN activity. Sci. Rep..

