# Supplementary material for: Development of a chub mackerel with less-aggressive fry stage by genome editing of arginine vasotocin receptor V1a2
Source: Sci Rep. 2023 Feb 23;13:3190. doi: 10.1038/s41598-023-30259-x (PMC9950132; doi:10.1038/s41598-023-30259-x)
Supplement: Supplementary file 1 — Supplementary Information. [file 41598_2023_30259_MOESM1_ESM.pdf]

Supplementary information

Development of a chub mackerel with less-aggressive fry stage by genome editing of arginine vasotocin receptor V1a2

Hirofumi Ohga[1], Koki Shibata[2], Ryo Sakanoue[2], Takuma Ogawa[2], Hajime Kitano[3], Satoshi Kai[2], Kohei Ohta[2], Naoki, Nagano[4], Tomoya Nagasako[5], Seiichi Uchida[5], Tetsushi Sakuma[6], Takashi Yamamoto[6], Sangwan Kim[7], Kosuke Tashiro[7], Satoru Kuhara[7], Koichiro Gen[8], Atushi Fujiwara[9], Yukinori Kazeto[10], Takanori Kobayashi[11] & Michiya Matsuyama[12*]

[1] Aqua-Bioresource Innovation Center (ABRIC) Karatsu satellite, Faculty of Agriculture, Kyushu University, Saga 847-0132, Japan.

[2] Laboratory of Marine Biology, Faculty of Agriculture, Kyushu University, Fukuoka 819-0395, Japan.

[3] Fishery Third Group, Marine Fisheries Research and Development Center, Japan Fisheries Research and Education Agency (FRA), Kanagawa, 221-8529, Japan.

[4] Laboratory of Aquaculture, Faculty of Agriculture, University of Miyazaki, Miyazaki 889-2192, Japan.

[5] Human Interface Laboratory, Factory of Information Science and Electrical Engineering, Kyushu University 819-0395, Fukuoka, Japan.

[6] Molecular Genetics Laboratory, Graduate School of Integrated Sciences for Life, Hiroshima University, Hiroshima 739-8526, Japan.

[7] Laboratory of Molecular Gene Technics, Faculty of Agriculture, Kyushu University, Fukuoka 812-8581, Japan.

[8] Planning and Coordination Department, Fisheries Technology Institute, FRA, Nagasaki 851-2213, Japan.

[9] Aquatic Breeding Division, Aquaculture Research Department, Fisheries Technology Institute, FRA, Mie 516-0193, Japan.

[10] Minamiizu Field Station, Fisheries Technology Institute, FRA, Shizuoka 415-0156, Japan.

[11] Aquatic Breeding Division, Aquaculture Research Department, Fisheries Technology Institute, FRA, Kanagawa 236-8648, Japan.

[12] ABRIC, Faculty of Agriculture, Kyushu University, Fukuoka 819-0395, Japan.

*Correspondence and requests for materials should be addressed to M.M. (email: rinya_m@agr.kyushu-u.ac.jp)

Computer software for video data analysis

The supplementary information describes the principle underlying the detection of abnormal behavior by image analysis software in detail.

Overview

The image analysis software is based on bioimage informatics technology, and automatically detects the data part where abnormal behavior was seen in the video. It took advantage of the habit of chub mackerel fry, which normally swim in groups. The image analysis software was programmed using the C ++ programming language.

The analysis software comprised several steps, as follows. First, it decomposed the video of 30 frames/s into a sequence of video frames. Second, it detected individual fry in the video frame. Third, it tracked the group of fry across frames to obtain the motion of each fry. Fourth, it represented the motion of the fry group in each video frame as a single feature vector. Till this step, we could have T feature vectors for T video frames. An hour (3,600 seconds)-long video generated 108,000 frames, and consequently, we had T = 108,000 vectors. Finally, using an anomaly detection method, called k-nearest neighbor (kNN), feature vectors showing some anomaly were detected. The number of the detected feature vectors indicates the number of anomalous behaviors, i.e., cannibalistic behaviors, in the video (Fig. 5b). Furthermore, in order to classify the detected abnormal behaviors as true cannibalistic behavior, collision with the wall, or others (such as panic state due to surprise), all detected frames were double-checked through visual inspection by human experts.

Fry detection in each frame

The location of individual fry was detected by combining two simple image-processing techniques, namely background subtraction and binarization, as shown in Supplementary Fig. S1a. The former considered the difference between a target video frame and a background image captured without the fry. Since the pool environment was static, background subtraction could enhance the region of individual fry. By taking the absolute value of difference for each pixel, a grayscale difference image was obtained. The latter was then applied to the difference image to determine the region of each fry. Finally, the location of the fry could be identified by the center of gravity of each fry region.

Tracking fry across frames

Fry were tracked in two consecutive frames. Among the various matching

algorithms, we used the stable marriage algorithm, which is a ranking-based matching algorithm between two sets (Supplementary Fig. S1b). While tracking 15 fry, for example, an individual (fry 1–15) detected in frame t would have a matching preference ranking based on its distance from an individual (fry # a–o) detected in the next frame $t+1$. The stable marriage algorithm searches for the most stable match, which would be the least inconvenient for this ranking. Tracking trajectories of the whole fry group were obtained by repeating this matching process for all consecutive frames (Supplementary Fig. S1c).

Representing the motions of fry as a feature vector

Based on the tracking results of each fry using the stable marriage algorithm, one feature vector representing the tracking results of all individuals was calculated in each frame. For this feature vector representation, the direction in which each fry swam (that is, the direction of movement) and the relevant speed (i.e., the distance traveled within the frame) were first quantized into 12 levels and four levels, respectively. The result was plotted in 48 bins of the 2D polar histogram, as shown in Supplementary Fig. S1d.

The 2D polar histogram was then modified to be "rotation-invariant" so that the motions shown in Supplementary Fig. S1d (a) and (b) have to be treated as similar. For example, in (a) and (b), the fry swam in a group in a certain direction, but the directions were opposite; therefore, the distance between the two frames increased. The pool was circular, and the absolute direction of motion in the pool was not important for detecting anomalous motions. Therefore, we made this histogram rotation-invariant by rotating the whole histogram so that the quantized direction with the most moves reached the top, as shown in Supplementary Fig. S1e. Using this operation, we could expect the motions shown in Supplementary Fig. S1d (a) and (b) to have very similar histograms. Next, we re-quantized the angle into four levels and obtained a 2D polar histogram with 16 bins (Supplementary Fig. S1e). Finally, the 16 elements of this histogram form a 16-dimensional vector at each frame.

Anomaly detection

By repeating the above process for all T frames, we generated 16-dimensional vectors in each frame. Our goal was to detect the vectors showing anomalous motions. The key idea of anomaly detection was that an anomalous motion becomes an "outlier," which is a vector very different from other vectors, as shown in Supplementary Fig. S1f. Vectors during cannibalistic behaviors are different from those of normal behaviors because cannibalistic behaviors often show very large and scattered motions of fry.

We employed kNN as an anomaly detection method. kNN evaluates the difference

between a target vector and its k-th neighboring vector. The difference represents the level of anomaly of the target vector. If $k = 1$, kNN evaluates the difference between a target vector and its nearest neighboring vector. However, the anomaly level by $k = 1$ is not suitable for our task, because motions are smooth along with frames even during a cannibalistic behavior and the motion vector of the next (or previous) frame becomes a very similar nearest-neighboring vector. In other words, we cannot find anomaly frames by $k = 1$. We, therefore, set k at a larger value to evaluate the levels of anomaly appropriately.

After calculating the anomaly level of all T vectors, we assumed the frames with the top 10% anomaly levels as “anomalies”. A cannibalistic behavior appears as consecutive anomaly frames. For example, in the analysis of this study, the average duration of one aggression was around 17 s (approximately 500 frames) in wild-type fry. We, therefore, removed short or isolated anomaly frames and then obtained the consecutive anomaly frames where cannibalistic behavior occurred.

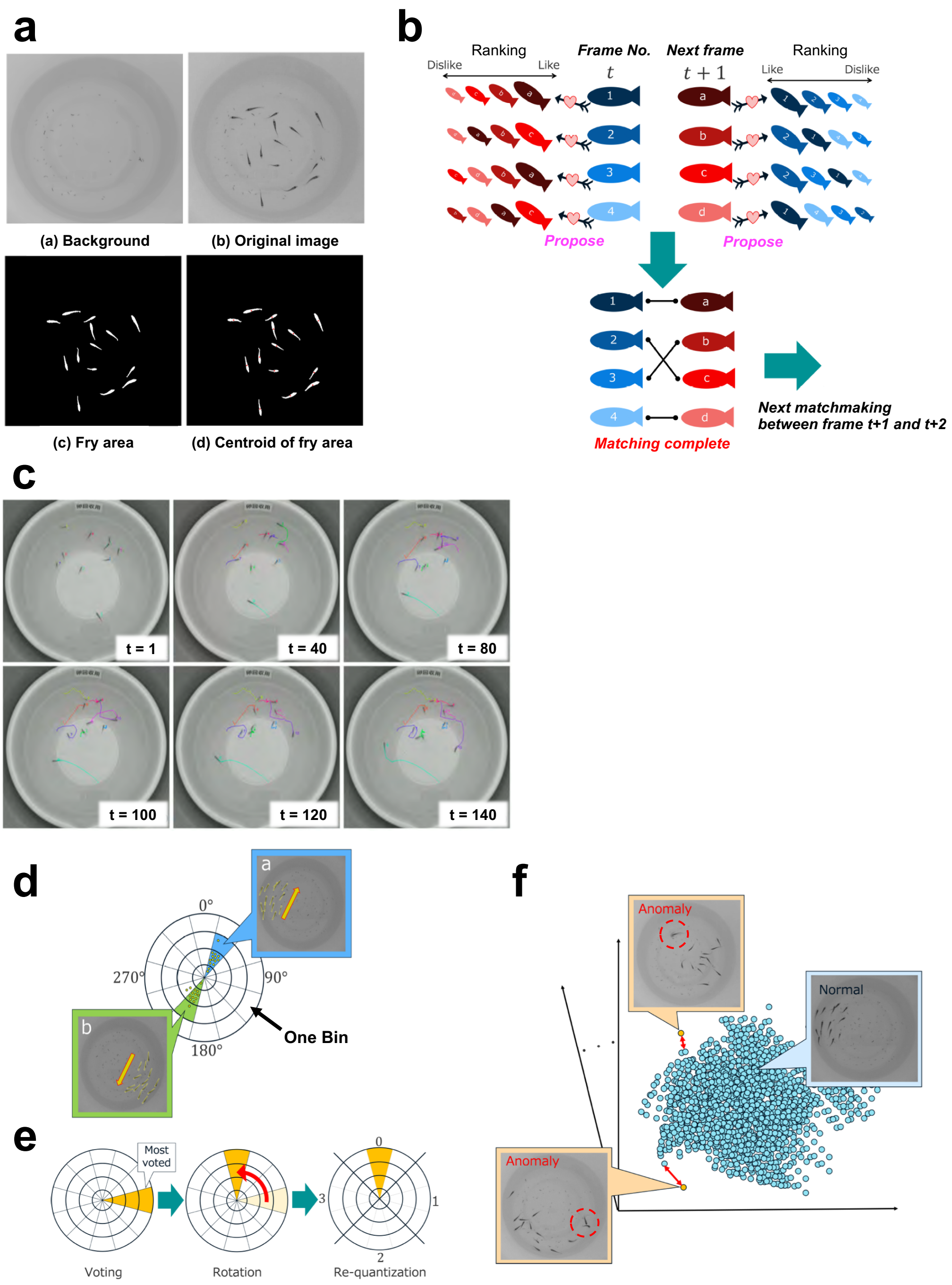

Fig. S1

Outline of the anomalous behavior detection algorithm. (a) Fry region extraction process. (b) Stable marriage algorithm. (c) Example of continuous tracking. (d) 48 bins of the 2D polar histogram. (e) Re-quantization of the vector's angle into 4 levels to get a 2D polar histogram with 16 bins. (f) Image of anomaly detection by k-nearest neighbor detection method.
